# Serum angiotensin-converting enzyme 2 as a potential biomarker for SARS-CoV-2 infection and vaccine efficacy

**DOI:** 10.3389/fimmu.2022.1001951

**Published:** 2022-10-12

**Authors:** Matthew P. Lennol, María-Salud García-Ayllón, Mariano Esteban, Juan García-Arriaza, Javier Sáez-Valero

**Affiliations:** ^1^ Instituto de Neurociencias de Alicante, Consejo Superior de Investigaciones Científicas (CSIC), Universidad Miguel Hernández, San Juan de Alicante, Spain; ^2^ Centro de Investigación Biomédica en Red sobre Enfermedades Neurodegenerativas (CIBERNED), San Juan de Alicante, Spain; ^3^ Unidad de Investigación, Fundación para el Fomento de la Investigación Sanitaria y Biomédica de la Comunitat Valenciana (FISABIO), Hospital General Universitario de Elche, Elche, Spain; ^4^ Department of Molecular and Cellular Biology, Centro Nacional de Biotecnología (CNB), Consejo Superior de Investigaciones Científicas (CSIC), Madrid, Spain; ^5^ Centro de Investigación Biomédica en Red de Enfermedades Infecciosas (CIBERINFEC), Madrid, Spain; ^6^ Instituto de Investigación Sanitaria y Biomédica de Alicante (ISABIAL), Alicante, Spain

**Keywords:** ACE2, serum, efficacy, biomarker, COVID-19, SARS-CoV-2, MVA-S vaccine

## Abstract

Various species of the SARS-CoV-2 host cell receptor, the angiotensin-converting enzyme 2 (ACE2), are present in serum, which may result from virus entry and subsequent proteolytic processing of the membrane receptor. We have recently demonstrated changes of particular ACE2 species in virus infected humans, either cleaved fragments or circulating full-length species. Here, we further explore the potential of serum ACE2 as a biomarker to test SARS-CoV-2 infection and vaccine efficacy in virus susceptible transgenic K18-hACE2 mice expressing human ACE2. First, in serum samples derived from K18-hACE2 mice challenged with a lethal dose of SARS-CoV-2, we observed an increase in the levels of cleaved ACE2 fragment at day 2 post-challenge, which may represent the subsequent proteolytic processing through virus entry. These elevated levels were maintained until the death of the animals at day 6 post-challenge. The circulating full-length ACE2 form displayed a sizable peak at day 4, which declined at day 6 post-challenge. Noticeably, immunization with two doses of the MVA-CoV2-S vaccine candidate prevented ACE2 cleaved changes in serum of animals challenged with a lethal dose of SARS-CoV-2. The efficacy of the MVA-CoV2-S was extended to vaccinated mice after virus re-challenge. These findings highlight that ACE2 could be a potential serum biomarker for disease progression and vaccination against SARS-CoV-2.

## Introduction

Angiotensin-converting enzyme 2 (ACE2) is the host cell receptor for the novel severe acute respiratory syndrome coronavirus 2 (SARS-CoV-2) that causes coronavirus disease 2019 (COVID-19). ACE2 is a ubiquitous glycoprotein abundantly expressed in humans in different tissues, which can also be found in plasma as several species that include circulating full-length forms and cleaved fragments (1).

Full-length ACE2 is a type I transmembrane glycoprotein with a short hydrophobic intracellular C-terminus (2). Shed fragments result from constitutive cleavage (2), but ACE2 is also cleaved after interaction with the SARS-CoV-2 spike (S) protein (3, 4). Indeed, binding of SARS-CoV-2 to the ACE2 receptor results in decreased abundance of ACE2 on the cell surface that can be attributed, at least in part, to enhanced shedding (5). Moreover, increased ACE2 levels have been found to be associated to several inflammatory processes including acute lung injury (6). ACE2 upregulation could also occur in SARS-CoV-2-infected tissues (7–9).

The existence of circulating full-length ACE2 forms, species that retain their transmembrane and intracellular domains, have been demonstrated in human plasma (1). Thus, cleaved ACE2 fragments and full-length ACE2 species co-exist in plasma and may be differentially affected during SARS-CoV-2 infection. Serial analysis of the different plasma ACE2 species at different time-points during the infection could also be informative as a read-out of the progression of the disease as well as therapy efficacy, including vaccination.

In this study, we monitored changes in different species of ACE2 in serum samples derived from susceptible K18-hACE2 transgenic mice infected with a lethal dose of SARS-CoV-2Click or tap here to enter text. We aimed to characterize and to determine the levels of co-existing ACE2 fragments and soluble full-length species using western blotting, a technique that allows the separation and quantification of individual ACE2 species. Elevated levels of the ACE2 fragments could reflect enhanced cleavage of the receptor triggered by SARS-CoV-2. Nevertheless, circulating ACE2 full-length forms can also increase, probably representing ACE2 tissue upregulation as a consequence of mass inflammation. Moreover, we analyzed serum ACE2 levels in K18-hACE2 mice immunized with the MVA-CoV2-S vaccine candidate and subsequently infected with SARS-CoV-2, which were fully protected against SARS-CoV-2 infection and mortality (10, 11). Noticeably, two doses of the MVA-CoV2-S vaccine candidate resulted in unaltered levels of serum ACE2. The efficacy of the vaccine candidate to prevent changes in serum ACE2 was also extended to mice re-challenged with a lethal dose of SARS-CoV-2. The results obtained demonstrate that the analysis of the levels of different ACE2 species could be a useful biomarker to estimate COVID-19 disease progression and vaccine efficacy.

## Material and methods

### Animals and ethics statement

Female transgenic K18-hACE2 mice, expressing the human ACE2 receptor, were obtained from The Jackson Laboratory (034860-B6.Cg-Tg(K18-ACE2)2Prlmn/J; genetic background C57BL/6J×SJL/J)F2). The efficacy experiments were previously described (10, 11) and were performed in the biosafety level 3 (BSL-3) facilities at the Centro de Investigación en Sanidad Animal (CISA)-Instituto Nacional de Investigaciones Agrarias (INIA-CSIC) (Valdeolmos, Madrid, Spain). The efficacy animal studies were approved by the Ethical Committee of Animal Experimentation (CEEA) of the CNB (Madrid, Spain) and by the Division of Animal Protection of the Comunidad de Madrid (PROEX 169.4/20 and 161.5/20). The specific study of the analysis of serum ACE2 in infected and immunized animals was also approved by the Ethics Committee of the Universidad Miguel Hernández and Departamento de Salud de Alicante - Hospital General (PI2020-063). Animal procedures conformed with international guidelines and with Spanish law under the Royal Decree (RD 53/2013).

### MVA-CoV2-S vaccine candidate

MVA-CoV2-S vaccine candidate against COVID-19 was used in this study, and its generation was previously described (10). MVA-CoV2-S (also termed MVA-S) expresses a human codon optimized full-length non-stabilized SARS-CoV-2 S protein from the Wuhan strain. Moreover, as a control we used the attenuated MVA-WT strain, obtained from the Chorioallantois vaccinia virus Ankara strain after 586 serial passages in chicken embryo fibroblast cells (12). All MVA viruses were grown in cultured chicken cells (DF-1), purified by centrifugation through two 36% (wt/vol) sucrose cushions in 10 mM Tris-HCl (pH 9) and tittered by immunostaining, as previously described (13).

### SARS-CoV-2 virus

SARS-CoV-2 strain MAD6 (kindly provided by José M. Honrubia and Luis Enjuanes, CNB-CSIC, Madrid, Spain) is a virus collected from a nasopharyngeal swab from a 69-year-old male COVID-19 patient from Hospital 12 de Octubre in Madrid (14). Growth and titration of SARS-CoV-2 MAD6 isolate has been previously described (10, 11). Full-length virus genome was sequenced, and it was found to be identical to SARS-CoV-2 reference sequence (Wuhan-Hu-1 isolate, GenBank: MN908947), except the silent mutation C3037>T, and two mutations leading to amino acid changes: C14408>T (in nsp12) and A23403>G (D614G in S protein).

### Efficacy study schedule in K18-hACE2 transgenic mice

For the SARS-CoV-2 infection study, female transgenic K18-hACE2 mice (19 weeks old, n= 11) were challenged with a lethal dose of 1×10^5^ plaque forming units (PFUs) of SARS-CoV-2 (MAD6 strain) by the intranasal route in 50 μl of PBS, as previously described (10, 11). Non-infected mice (n= 9) were used as a control group. Mice were monitored for body weight change and mortality; animals with more than a 25% of weight loss were euthanized. Due to this body weight loss, all K18-hACE2 mice challenged with SARS-CoV-2 were sacrificed 6 days post-challenge (10). At days 2 (n= 3 in infected mice; n= 2 in non-infected mice), 4 (n= 3 in infected mice; n= 2 in non-infected mice) and 6 (n= 5 in infected mice) post-challenge mice were sacrificed, submandibular blood was collected and serum samples were thereafter obtained.

For the immunization study, female K18-hACE2 mice (10 weeks old) immunized with one or two doses of MVA-CoV2-S were used to evaluate the efficacy of the MVA-CoV2-S vaccine candidate, as previously described (10, 11). Groups of animals (n= 11) received one or two doses of 1×10^7^ PFUs of MVA-CoV2-S by intramuscular route in 100 μl of PBS (50 μl/leg) at 0 and 4 weeks. Mice primed and boosted with non-recombinant MVA-WT were used as a control group. At week 9, mice were challenged with the lethal dose (1×10^5^ PFUs) of SARS-CoV-2 MAD6 strain. Submandibular blood collection to obtain serum samples was performed at days 2 (n= 3/group), 4 (n= 3/group) and 15 (n= 5/group) after virus challenge.

Moreover, K18-hACE2 mice previously vaccinated with one or two doses of MVA-CoV2-S (or control unvaccinated and unchallenged mice) (n= 5/group) were re-infected with SARS-CoV-2 at week 16 (7 weeks after the first SARS-CoV-2 infection) with the lethal dose (1×10^5^ PFUs) of SARS-CoV-2 MAD6 strain by intranasal route in 50 μl of PBS. Mice were monitored for body weight change and mortality for 6 days post-rechallenge, moment at which control infected mice lost more than 25% of their initial body weight and had to be sacrificed. Thus, at day 6 post-rechallenge, all mice (n= 5/group) were euthanized, blood was collected and serum samples were obtained.

### Detection of ACE2 in serum samples by quantitative fluorescent western blotting

Blood was collected by submandibular bleeding, maintained at 37°C for 1 hour, kept at 4°C overnight, and centrifuged at 3600 rpm for 20 min at 4°C to obtain the serum samples, which were then inactivated at 56°C for 30 min and kept at -80°C until use. ACE2 species were detected by fluorescent-based imaging after sodium dodecyl sulfate-polyacrylamide gel electrophoresis (SDS-PAGE) and western blotting. This technique provides a wider linear dynamic range than chemiluminescent detection (15) including a greater upper linear range of detection (16). Serum samples were heated in reducing Laemmli SDS sample buffer (Thermo ScientificTM) for 7 min at 70°C (dilution ratio 1:10). Serum samples (0.6 μl loaded) were then resolved on 7.5% SDS-PAGE gels (Mini-PROTEAN^®^ TGX™ Precast Gels; Bio-Rad) and transferred to 0.2 μm nitrocellulose membranes (Bio-Rad). Then, the membrane was blocked with Odyssey Blocking Buffer (PBS) and incubated with the anti-ectodomain AF933 antibody (1:200 dilution) or alternatively with the anti-C-terminus ab15348 antibody (1:500 dilution). Finally, blots were washed and incubated with the appropriate conjugated secondary antibodies (IRDye 800CW donkey anti-goat and IRDye 680 RD goat anti-rabbit, LI-COR Biosciences) and imaged on an Odyssey Clx Infrared Imaging System (LI-COR Biosciences). For quantitative analysis all blots were resolved with the AF933 antibody, which resolves all species, while the ab15348 antibody was used to define the truncated C-terminal fragments. Band intensities were analyzed using LI-COR software (Image Studio Lite). To estimate the relative ratio of ACE2 species for each sample, the immunoreactivity was considered for each of the bands (see Results section).

### Statistical analysis

All data were analyzed using SigmaStat (Version 3.5; SPSS Inc.). The Kolmogorov-Smirnov test was used to analyze the distribution of each variable. ANOVA was used for parametric variables and the Kruskal-Wallis test for non-parametric variables for comparison between groups. A Student’s t-test for parametric variables and a Mann-Whitney U test for non-parametric variables were employed for comparison between two groups, and for determining *p* values. The results are presented as means ± standard error of the mean (SEM).

## Results

### SARS-CoV-2 virus causes changes in ACE2 plasma species in infected K18-hACE2 transgenic mice

K18-hACE2 mice were challenged with a lethal intranasal dose of SARS-CoV-2 (MAD6 isolate, 1×10^5^ PFUs/mouse). Changes in body weight and mortality after SARS-CoV-2 infection were previously reported and showed that all infected mice lost more than 25% of their body weight and were sacrificed at 6 days post-challenge (10). To define the impact of SARS-CoV-2 infection on the levels of circulating ACE2 species serum samples obtained in non-infected animals (day 0) and at days 2, 4 and 6 post-infection were analyzed.

**Figure 1:**
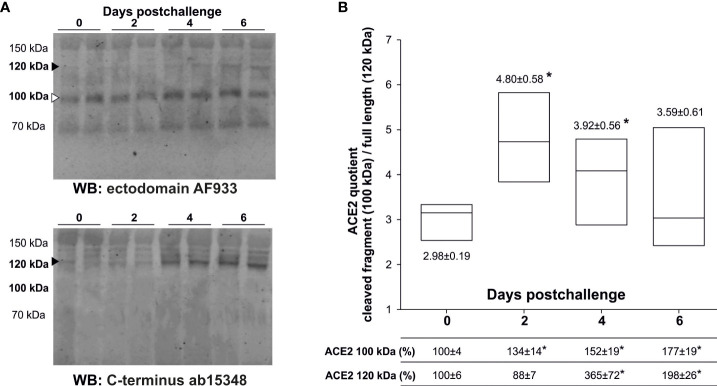
Levels of serum ACE2 species from challenged K18-hACE2 mice. Transgenic K18-hACE2 mice (n= 11), expressing human ACE2, were challenged with a lethal dose of SARS-CoV-2 (MAD6 strain) by the intranasal route. Serum ACE2 species were analyzed in non-infected mice (day 0; n= 9), and at days 2 (n= 3), 4 (n= 3) and 6 (n= 5) postchallenge. **(A)** Representative western blots with two different cases for each condition are showed; resolved with the ectodomain AF933 and the C-terminus ab15348 antibodies to define cleaved fragments (white arrow-head) and full-length (black arrow-head) species. The cleaved 100 kDa ACE2 species after shedding are recognized by the AF933, but not by the C-terminal ab15348 antibody. **(B)** The densitometric quantification of the full-length 120 kDa ACE2 species (black arrow-head) and its presumable cleaved 100 kDa fragment (white arrow-head) were estimated from the immunoreactivity obtained with the AF933 antibody, and as the percentage respect to day 0 levels. The densitometric quantification of the 120 kDa ACE2 species obtained with the ab15348 is presented in the main text. All samples were analyzed at least in duplicate in separate blots. Box plots represent the quotient obtained by dividing the level of immunoreactivity of the 100 kDa fragment by the level of immunoreactivity of the 120 kDa full-length form. The bars within the box plot represent the median abundance for the given group. (*) significant *p* values (*p*< 0.05) respect to day 0.

In a previous report, we demonstrated the presence of several soluble ACE2 full-length species as well as cleaved fragments in human plasma and in serum samples from K18-hACE2 mice (1). Full-length ACE2 is 805 amino acids-long protein with an apparent molecular mass of ~100-130 kDa (2), while shedding results in ~20 kDa smaller fragments than the original full-length species (1). Here, we analyzed serum samples from infected K18-hACE2 mice by western blotting using either a polyclonal goat antibody (AF933) that recognizes the ectodomain of ACE2, or a rabbit polyclonal antibody (ab15348) raised against the C-terminus of human ACE2, and confirmed the existence of several immunoreactive bands ranking from 70 to 150 kDa (**Figure 1A**). These antibodies also recognize the murine ACE2 species present in C57BL/6J wild-type mice (blots not shown). The bands uniquely immunoreactive to the ectodomain antibody that are not recognized by the C-terminal antibody should represent C-terminally truncated shed fragments (1). To follow changes in ACE2 processing we focused the analysis on the 120 kDa full-length and the 100 kDa truncated species, the last being compatible in molecular mass with the expected shed fragment originated from the 120 kDa species (**Figure 1B**). Remarkably, only the 100 kDa ACE2 cleaved fragment, estimated with the AF933 antibody, was increased at day 2 post-challenge, compared to levels at day 0 (134 ± 14%; *p*= 0.024). We interpreted that this change represents the virus entry and subsequent proteolytic processing of the membrane receptor, whose full-length form displayed a non-significant decrease, resulting in increased levels of the ACE2 “cleaved/full-length” quotient. However, at day 4 post-challenge the ACE2 species identified as full-length forms displayed a large increase (365 ± 72%; *p*= 0.023), two times higher than the change present in the cleaved fragment, causing a decrease in the quotient. This change was validated with the C-terminal antibody ab15348 (407 ± 104% respect to non-infected animals; *p*= 0.03). This large increase in the levels of the ACE2 full-length forms probably represents ACE2 upregulation as a consequence of massive inflammation and/or progression of cell death. At 6 days post-challenge, prior to the sacrifice of the animals, plasma ACE2 remained elevated (AF933 antibody: 177 ± 19%; *p*= 0.004), but less so than at 4 days post-challenge, suggesting that the mechanism related to ACE2 release in plasma is not cell death, which should increase progressively. Once again, the change was validated with the ab15348 antibody (188 ± 20%; *p*= 0.006).

### MVA-CoV2-S vaccine candidate prevents plasma ACE2 changes induced by SARS-CoV-2 virus in infected K18-hACE2 transgenic mice

**Figure 2:**
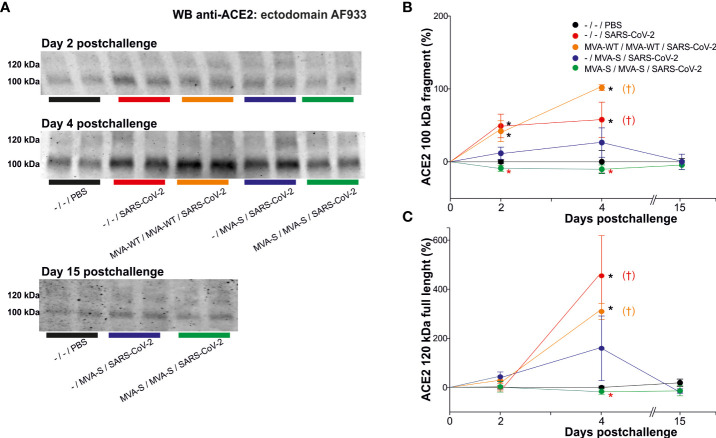
MVA-CoV2-S vaccine candidate prevented serum ACE2 changes. K18-hACE2 transgenic mice (n= 11/group) were immunized by the intramuscular route with one or two doses of 1×10^7^ PFUs of MVA-CoV2-S (also termed MVA-S) at weeks 0 and 4. Then, at week 9, mice were challenged intranasally with a lethal dose of SARS-CoV-2 (MAD6 isolate; 1×10^5^ PFUs). MVA-WT-inoculated and unvaccinated **(-)** mice (n= 11) were also intranasally challenged with SARS-CoV-2. Unvaccinated and unchallenged mice (n= 9) were used as a negative control (PBS). At days 2 (n= 3/group), 4 (n= 3/group), and 15 (n= 5) serum was collected. **(A)** Representative ACE2 immunoblots for samples at indicated situation resolved with the ectodomain AF933 antibody. Densitometric quantified for the cleaved 100 kDa fragment **(B)** and for the full-length 120 kDa ACE2 species **(C)**. All samples were analyzed at least in duplicate in separate blots. †: All mice inoculated with MVA-WT or unvaccinated and infected were sacrificed at 6 days post-challenge. Significant *p* values (*p*< 0.05) respect to unvaccinated and unchallenged mice (*; -/-/PBS), or respect to unvaccinated and challenged mice (*; -/-/SARS-CoV-2).

Previous studies in K18-hACE2 transgenic mice demonstrated the full efficacy against SARS-CoV-2 infection triggered by the MVA-CoV2-S vaccine candidate (also termed MVA-S) with a significant reduction in viral load, histopathology and pro-inflammatory cytokine expression levels in lung samples (10, 11, 17). Here, we evaluated if the analysis of serum ACE2 levels in vaccinated and challenged mice corroborated the efficacy of MVA-CoV2-S and served to assess if MVA-CoV2-S prevents virus entry/infection in the cells (**Figure 2A**). K18-hACE2 mice were intramuscularly immunized at weeks 0 and 4 with two doses of MVA-CoV2-S or at week 4 with one dose of MVA-CoV2-S, and then challenged 5 weeks later with a lethal dose of SARS-CoV-2 as indicated above. Challenged mice primed and boosted with MVA-WT, unvaccinated mice, and unchallenged mice were all used as control groups. Our previous reports showed that all mice vaccinated with two doses of MVA-CoV2-S did not lose body weight post-challenge and survived, whereas mice immunized with one dose of MVA-CoV2-S lost body weight during the first 4 days post-challenge, but they recovered and survived (10, 11, 17). Here, we showed that at day 2 post-challenge the increased levels of the ACE2 cleaved fragment detected in unvaccinated infected K18-hACE2 mice was prevented in mice immunized with two doses of MVA-CoV2-S (**Figure 2B**), while there were no differences in the full-length form (**Figure 2C**). Moreover, at day 4 post-challenge the increase in both the ACE2 cleaved fragment and full-length species detected in unvaccinated K18-hACE2 infected mice were also prevented in mice vaccinated with two doses of MVA-CoV2-S (**Figures 2B, C**). The unaltered levels of the full-length were again corroborated with the ab15348 antibody (blots not shown). Levels of the ACE2 cleaved fragment and full-length species at day 4 post-challenge in mice vaccinated with one dose of MVA-CoV2-S were intermediate between unvaccinated infected mice and mice vaccinated with the two-dose regimen (**Figures 2B, C**), coinciding with the loss of body weight observed in mice vaccinated with one dose of MVA-CoV2-S at 4 days post-challenge (10, 11, 17), thus reflecting that one dose of MVA-CoV2-S was less efficacious at this time point than two doses. At 6 days post-challenge unvaccinated animals were sacrificed. We next analyzed the levels of ACE2 cleaved fragment and full-length species in all vaccinated mice at day 15 post-challenge (**Figure 2A**), and the results showed that these levels were similar to those seen in uninfected mice (**Figures 2B, C**).

**Figure 3:**
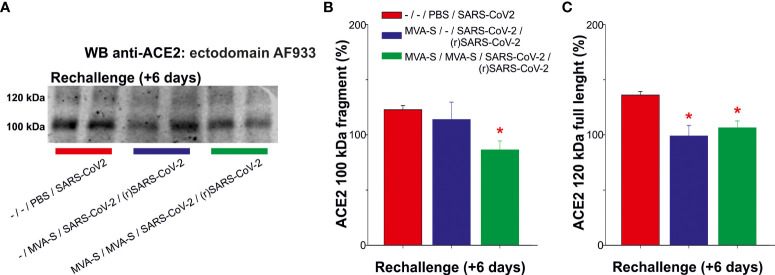
Outcome of SARS-CoV-2 reinfection in serum ACE2 levels of MVA-CoV2-S vaccinated K18-hACE2 mice that recovered from a first virus infection. To determine the effect of a second virus inoculation, mice vaccinated with one or 2 doses of MVA-CoV2-S (n= 5/group), were rechallenged with the lethal dose of SARS-CoV-2, 7 weeks after the first SARS-CoV-2 infection. Unvaccinated mice (n= 5) were also challenged with the virus, dying at day 6 post-challenge. Individual mouse serum samples collected 6 days after re-challenge [(r)SARS-CoV-2] were analyzed. **(A)** Representative ACE2 immunoblot for samples at indicated situation resolved with the ectodomain AF933 antibody. **(B, C)** Densitometric quantified for the cleaved 100 kDa fragment **(B)** and for the full-length 120 kDa **(C)** species. All samples were analyzed at least in duplicate in separate blots. Significant *p* values (*p*< 0.05) respect to unvaccinated and challenged mice (*; -/-/PBS/SARS-CoV-2).

Previous studies also demonstrated that K18-hACE2 transgenic mice vaccinated with one or two doses of MVA-CoV2-S are fully protected against SARS-CoV-2 reinfection (11). Therefore, all control and vaccinated mice that survived the first SARS-CoV-2 infection were re-challenged with the lethal dose of SARS-CoV-2 (1×10^5^ PFU/mouse). The control unvaccinated and challenged mice lost body weight and died at 6 days post-challenge. The analysis of serum samples at day 6 post-reinfection showed that two doses of MVA-CoV2-S prevented the occurrence of elevated ACE2 levels of cleaved fragment and of full-length species (**Figure 3**). The increase in full-length ACE2 displayed for unvaccinated mice was also nullified in one dose-vaccinated mice, which only displayed elevated levels of the cleaved fragment (**Figure 3**).

## Discussion

Receptor recognition by SARS-CoV-2 is an important determinant of viral infectivity. The subsequent proteolytic processing of ACE2, as well potential changes in ACE2 expression levels, could be relevant read-outs of infection progression and of therapeutic effectivity. However, it is not clear how altered ACE2 species serum levels reflect SARS-CoV-2 virulence. Fragments and proteolytic unprocessed full-length ACE2 species can be present as soluble species, reflecting different outcomes for the pathology. In fact, we have demonstrated the co-existence of ectodomain fragments (C-terminal truncated) and soluble full-length species in human plasma and transgenic K18-hACE2 mice serum (1).

In the plasma of COVID-19 patients suffering a moderate presentation of the disease, the ACE2 full-length species decreased, while a truncated fragment was marginally higher during the acute phase of infection (1). The elevated levels of the ACE2 fragment triggered by SARS-CoV-2 can reflect enhanced receptor interaction, internalization and further proteolytic cleavage (18). It is also plausible that soluble circulating full-length species reflect the tissue content of ACE2. The cleaved ACE2 fragments would increase at the expense of membrane-resident ACE2. In this context, it has been reported that in acute maternal SARS-CoV-2 infections the increase in maternal serum ACE2 may be the result of ACE2 shedding, since villous placental tissues displayed decreased ACE2 protein levels despite ACE2 a remarkable increase in mRNA expression (19). Here, we found that the early change detected in plasma from SARS-CoV-2 challenged K18-hACE2 mice is the result of elevated levels of truncated ACE2 fragments, probably reflecting increased membrane shedding triggered by the virus entry.

A few studies have reported that total serum ACE2 levels are elevated in severe (20) and in critically ill COVID-19 patients (21), with higher levels of serum ACE2 occurring in severe cases (22) and in non-survivor infected individuals (23). Indeed, increased plasma ACE2 levels from early to late stages of the disease have been reported in severe COVID-19 patients (24), and an upward trajectory of circulating ACE2 is associated with an elevated risk of mortality and organ injuries (25). In a previous study, total plasma ACE2 levels remained elevated following SARS-CoV-2 infection (26), while another study indicated only transient elevated ACE2 levels that decreased during the course of infection (27); these discrepancies may be related with the severity of the disease. Besides, the elevated ACE2 levels in infected individuals decreases at follow-up in recovered subjects (28). Most of these studies also concluded that circulating ACE2 should appear elevated in COVID-19 patients due to increased shedding from infected cells. An increase in circulating extracellular vesicles that express ACE2 in the plasma of COVID-19 patients has been also demonstrated (29). It is relevant to note that other studies of total plasma ACE2 levels failed to report changes in SARS-CoV-2 infected individuals (30–33) or to discriminate levels regarding the prognosis or mortality of COVID-19 patients (34). Lower plasma concentrations of ACE2 in COVID-19 patients compared with healthy controls has also been reported in prolonged viral shedders (35), and recently decreased plasma ACE2 activity was detected in an infected individual, but showed an inverse correlation with ACE2 protein concentration (36). These discrepancies can be interpreted partially due to handling of the samples, interference of endogenous inhibitors, presence of active and inactive forms, but also due to the fact that most of the recent studies on circulating ACE2 levels in SARS-CoV-2 infected subjects are mainly studies based on the determination of ACE2 enzymatic activity or ELISA assays. These methodological approaches do not discriminate between fragments and proteolytic unprocessed full-length ACE2 species, or potential different dynamics during disease progression. Anyhow, further studies addressing ACE2 levels of the different species and their enzymatic activity levels during SARS-CoV-2 infection and treatment are of interest for the potential metabolic impact in affected individuals, since imbalances in the renin-angiotensin system have been implicated in modulation of inflammation (37) and in several pathological conditions such as acute respiratory distress syndrome (38). In fact, different dynamics in full-length and cleaved ACE2 species may overlap during disease progression, thus hindering the interpretation of the progression of virus infection. Particularly, the contribution of the unprocessed full-length species that would mirror tissue changes during viral infection may differ in mild and severe COVID-19 affected patients. As mentioned above, our previous study indicated that patients with moderate COVID-19 in the acute phase of infection had significantly decreased levels of full-length ACE2 species, and the levels returned to the normal range in patients after a recovery period (1). Decreases in circulating full-length ACE2 can reflect the decrease of these species in the tissue due to enhanced shedding of the membrane-resident ACE2, but could also reflect altered expression levels. Early studies with SARS-CoV-1 indicated that in mice lung injury appeared to be mediated by downregulation of ACE2 expression (39). However, analysis of gene expression data from cells in bronchoalveolar lavage fluid from COVID-19 patients suggest that ACE2 becomes upregulated during SARS-CoV-2 infection (8). It has been reported that ACE2 protein expression is significantly higher in COVID-19 post-mortem lung tissues than in non-COVID-19 patients for other indications (40). Upregulated ACE2 expression in SARS-CoV-2-infected tissues has been associated to an excessive immune response to SARS-CoV-2 infection (7), suggesting that ACE2 might regulate the immune response through immunological activation-associated pathways in the process of SARS-CoV-2 infection (9). Thus, we cannot discard that changes in ACE2 gene expression may be differently regulated over time during infection and in a different manner in infected subjects suffering an excessive immune response to SARS-CoV-2. In this study, a lethal dose of SARS-CoV-2 in transgenic K18-hACE2 mice probably results in increased levels of circulating full-length ACE2 species driven by the upregulation at tissular levels caused by an exacerbated immune-inflammatory response that occurred after virus entry, monitored by an early increase in circulating ACE2 fragments. In this context, plasma ACE2 levels have been found to be increased in several inflammatory processes, including renal and cardiovascular disease (41, 42) and acute lung injury (6). Elevated plasma ACE2 levels have been associated with increased levels of the pro-inflammatory interleukin-6 levels in severe COVID-19 disease (24), while the anti-inflammatory interleukin-13 IL-13 modulates ACE2 expression in airway epithelial cells in asthma and atopy (43); altogether these results support a link between ACE2 and inflammation. Understanding the mechanism of the release of the full-length membrane-bound form of ACE2 into biological fluids will be of great value for interpreting plasma ACE2 changes as a read-out of disease progression.

Remarkably, we have demonstrated that the analysis of the circulating ACE2 species indicate that two doses of the MVA-CoV2-S vaccine candidate prevented SARS-CoV-2 entry to the cell, since the increased generation of cleaved fragment was avoided during the first virus challenge, and also after virus re-challenge. Our previous study demonstrated that two doses of the MVA-CoV2-S vaccine candidate fully protected K18-hACE2 transgenic mice from a lethal dose of SARS-CoV-2, while mice immunized with one single dose of MVA-CoV2-S and challenged with SARS-CoV-2 lost body weight during the first days post-challenge, but by day 4 they all recovered and survived (10). Interestingly, the levels of ACE2 fragments from mice immunized with one single dose of MVA-CoV2-S show a trend to decrease compared with levels determined in unvaccinated mice, but the change resulted non-significant, probably indicating that one dose is not as effective as two doses in preventing SARS-CoV-2 entry to the cells (and subsequent proteolytic processing of the membrane receptor).

Assuming that the increase in circulating full-length ACE2 detected in infected unvaccinated mice is related with an exacerbated immune-inflammatory response that follows SARS-CoV-2 infection and tissue replication, two doses of MVA-CoV2-S fully prevent an increase in ACE2 levels compared to unvaccinated mice, while one single dose of MVA-CoV2-S only prevents changes at re-challenge, again demonstrating that two doses of MVA-CoV2-S drive an early and robust immunogenicity and efficacy.

This study has several limitations. In this regard, altered ACE2 serum levels were not confronted with analysis of particular tissues, and we assumed, but did not probe, whether ACE2 full-length species reflect tissue content. Moreover, the translation to humans, where serum ACE2 exhibits a more complex pattern of bands, probably reflecting different tissular origins, appears difficult, in which severe cases may vary regarding the affectation of particular tissues. Moreover, our previous study in human samples (1) suggest a broad range of plasma ACE2 levels existing within the general population, since ACE2 expression and shedding can be altered in many common conditions such as hypertension, heart disease, diabetes or obesity, among many others. Furthermore, it would be interesting to validate our findings within alternative trials with other vaccines, and by independent laboratories. In this regard, most of the laboratories that reported altered ACE2 levels during SARS-CoV-2 infection addressed the investigation by desirable approaches for quantitative analysis such as ELISA or enzymatic activity, but these methods do not detect subtle changes in specific ACE2 species, reflecting specific outcomes of processing.

Overall, here we demonstrate that the determination of ACE2 cleaved fragments and full-length circulating species are potential biomarkers with value for reinforcing the translation of MVA-CoV2-S and other therapeutic interventions to clinical trials. Moreover, these ACE2 species, both full-length and cleaved, might be relevant serum markers to predict the effectiveness of SARS-CoV-2 vaccines.

## Data availability statement

The raw data supporting the conclusions of this article will be made available by the authors, without undue reservation.

## Ethics statement

The animal study was reviewed and approved by Ethics Committee of the Universidad Miguel Hernández, Departamento de Salud de Alicante - Hospital General, Hospital General Universitario de Alicante, Ethical Committee of Animal Experimentation (CEEA) of the CNB (Madrid, Spain), and Division of Animal Protection of the Comunidad de Madrid.

## Author contributions

Conceptualization: JG-A and JS-V. Formal analysis: ML and M-SG-A. Funding acquisition: ME, JG-A, and JS-V. Investigation: ML, M-SG-A, and JG-A. Methodology: ML, M-SG-A, and JG-A. Resources: ME and JG-A. Supervision: JG-A and JS-V. Validation: JG-A and JS-V. Visualization: JG-A and JS-V. Writing original draft: JS-V. Writing, review and editing: all authors. All authors contributed to the article and approved the submitted version.

## Funding

This study was funded in part by the Instituto de Investigación Sanitaria y Biomédica de Alicante (ISABIAL; grant 2020-0308), the Direcció General de Ciència I Investigació, Generalitat Valenciana (AICO/2021/308), and by the Instituto de Salud Carlos III (ISCIII, grants PI19-01359), co-financed by the Fondo Europeo de Desarrollo Regional (FEDER, “Investing in your future”) and through CIBERNED, ISCIII. We also acknowledge financial support from the Spanish Ministerio de Economía y Competitividad, through the “Severo Ochoa” Programme for Centres of Excellence in R&D (SEV-2017-0723). This research was also supported by Fondo COVID-19 grant COV20/00151 (Spanish Health Ministry, Instituto de Salud Carlos III (ISCIII)), Fondo Supera COVID-19 grant (Crue Universidades-Banco Santander), and Spanish Research Council (CSIC) grant 202120E079 (to JG-A); CSIC grant 2020E84, Ferrovial, and MAPFRE donations (to ME); and Spanish Ministry of Science and Innovation (MCIN)/Spanish Research Agency (AEI)/10.13039/501100011033 grant (PID2020-114481RB-I00; to JG-A and ME). This research work was also funded by the European Commission- NextGenerationEU, through CSIC’s Global Health Platform (PTI Salud Global) (to JG-A and ME). MPL is supported by a BEFPI scholarship from the Generalitat Valenciana. JGA and ME acknowledges financial support from the Spanish State Research Agency, AEI/10.13039/501100011033, through the “Severo Ochoa” Programme for Centres of Excellence in R&D (SEV-2013-0347, SEV-2017-0712).

## Acknowledgments

We thank Centro de Investigación en Sanidad Animal (CISA)-Instituto Nacional de Investigaciones Agrarias (INIA-CSIC) (Valdeolmos, Madrid, Spain) for the BSL-3 facilities. SARS-CoV-2 MAD6 virus isolate was kindly provided by Drs. José M. Honrubia and Luis Enjuanes (CNB-CSIC, Madrid, Spain).

## Conflict of interest

The authors declare that the research was conducted in the absence of any commercial or financial relationships that could be construed as a potential conflict of interest.

## Publisher’s note

All claims expressed in this article are solely those of the authors and do not necessarily represent those of their affiliated organizations, or those of the publisher, the editors and the reviewers. Any product that may be evaluated in this article, or claim that may be made by its manufacturer, is not guaranteed or endorsed by the publisher.
